# Synaptic configuration of quadrivalents and their association with the XY bivalent in spermatocytes of Robertsonian heterozygotes of *Mus domesticus*

**DOI:** 10.1186/s40659-017-0143-6

**Published:** 2017-11-23

**Authors:** Soledad Berríos, Raúl Fernández-Donoso, Eliana Ayarza

**Affiliations:** 1 Programa Genética Humana, ICBM, Facultad de Medicina, Universidad de Chile, Independencia No 1027, P.O.Box 70061, Santiago 7, Chile; 2 Departamento de Tecnología Médica, Facultad de Medicina, Universidad de Chile, Santiago, Chile

**Keywords:** Quadrivalents, Robertsonian chromosomes, Mouse spermatocytes, Nucleoli, Nuclear architecture

## Abstract

**Background:**

The nuclear architecture of meiotic prophase spermatocytes is based on higher-order patterns of spatial associations among chromosomal domains and consequently is prone to modification by chromosomal rearrangements. We have shown that nuclear architecture is modified in spermatocytes of Robertsonian (Rb) homozygotes of *Mus domesticus*. In this study we analyse the synaptic configuration of the quadrivalents formed in the meiotic prophase of spermatocytes of mice double heterozygotes for the dependent Rb chromosomes: Rbs 11.16 and 16.17.

**Results:**

Electron microscope spreads of 60 pachytene spermatocytes from four animals of *Mus domesticus* 2n = 38 were studied and their respective quadrivalents analysed in detail. Normal synaptonemal complex was found between arms 16 of the Rb metacentric chromosomes, telocentrics 11 and 17 and homologous arms of the Rb metacentric chromosomes. About 43% of the quadrivalents formed a synaptonemal complex between the heterologous short arms of chromosomes 11 and 17. This synaptonemal complex is bound to the nuclear envelope through a fourth synapsed telomere, thus dragging the entire quadrivalent to the nuclear envelope. About 57% of quadrivalents showed unsynapsed single axes in the short arms of the telocentric chromosomes. About 90% of these unsynapsed quadrivalents also showed a telomere-to-telomere association between one of the single axes of the telocentric chromosome 11 or 17 and the X chromosome single axis, which was otherwise normally paired with the Y chromosome. Nucleolar material was associated with two bivalents and with the quadrivalent.

**Conclusions:**

The spermatocytes of heterozygotes for dependent Rb chromosomes formed a quadrivalent where four chromosomes are synapsed together and bound to the nuclear envelope through four telomeres. The nuclear configuration is determined by the fourth shortest telomere, which drags the centromere regions and heterochromatin of all the chromosomes towards the nuclear envelope, favouring the reiterated encounter and eventual rearrangement between the heterologous chromosomes. The unsynapsed regions of quadrivalents are frequently bound to the single axis of the X chromosome, possibly perturbing chromatin condensation and gene expression.

**Electronic supplementary material:**

The online version of this article (10.1186/s40659-017-0143-6) contains supplementary material, which is available to authorized users.

## Background

Rb translocations involve double-strand DNA breaks of the centromere in two telocentric (acrocentric) chromosomes followed by repair (fusion) ligating the respective long arms and creating a metacentric Rb chromosome [1]. Rb translocations are frequently present in natural populations of the house mouse *Mus musculus domesticus*. As a consequence, the standard diploid karyotype of the house mouse consisting of 40 telocentric chromosomes may be reduced by the emergence of metacentric Rb chromosomes. This natural process has produced more than 40 different chromosomal races, ranging from 2n = 40 to 2n = 22 [2]. Two distinct types of Rb heterozygotes have been recognized: (1) complex heterozygotes which carry two or more metacentric chromosomes with common chromosome arms that consequently form rings or chains of more than three chromosomes at meiosis I and (2) simple or multiple simple heterozygotes which carry one or more metacentric chromosomes and homologous telocentrics that form trivalents during meiosis I [3, 4].

We have shown that Rb chromosomes change the nuclear architecture of spermatocytes in meiotic prophase. This means that the chromosomal domains of the derived Rb chromosome have a different location in the nuclear space of the spermatocyte, and also changes the probability of interaction between chromosomal domains of non-homologous chromosomes [5]. In this study we were interested in the synaptic configuration of chromosomes involved in the complex heterozygote 2n = 38 that carry two metacentric Rb chromosomes that share two chromosome arms (Rbs: 11.16 and 16.17), as a way of understanding the meiotic origin of reduced fertility in these hybrids. The electron microscopy of 60 micro spreadings of pachytene spermatocytes confirmed the organization of quadrivalents integrated by chromosomes 11, 11.16, 16.17 and 17. The synaptic configuration of the quadrivalent and the frequent associations between an unsynapsed axis of a telocentric chromosome and the XY bivalent are described. We propose a mechanism for this kind of heterologous association and discuss the nuclear configurations of quadrivalents in associated and non-associated conditions.

## Results

**Fig. 1:**
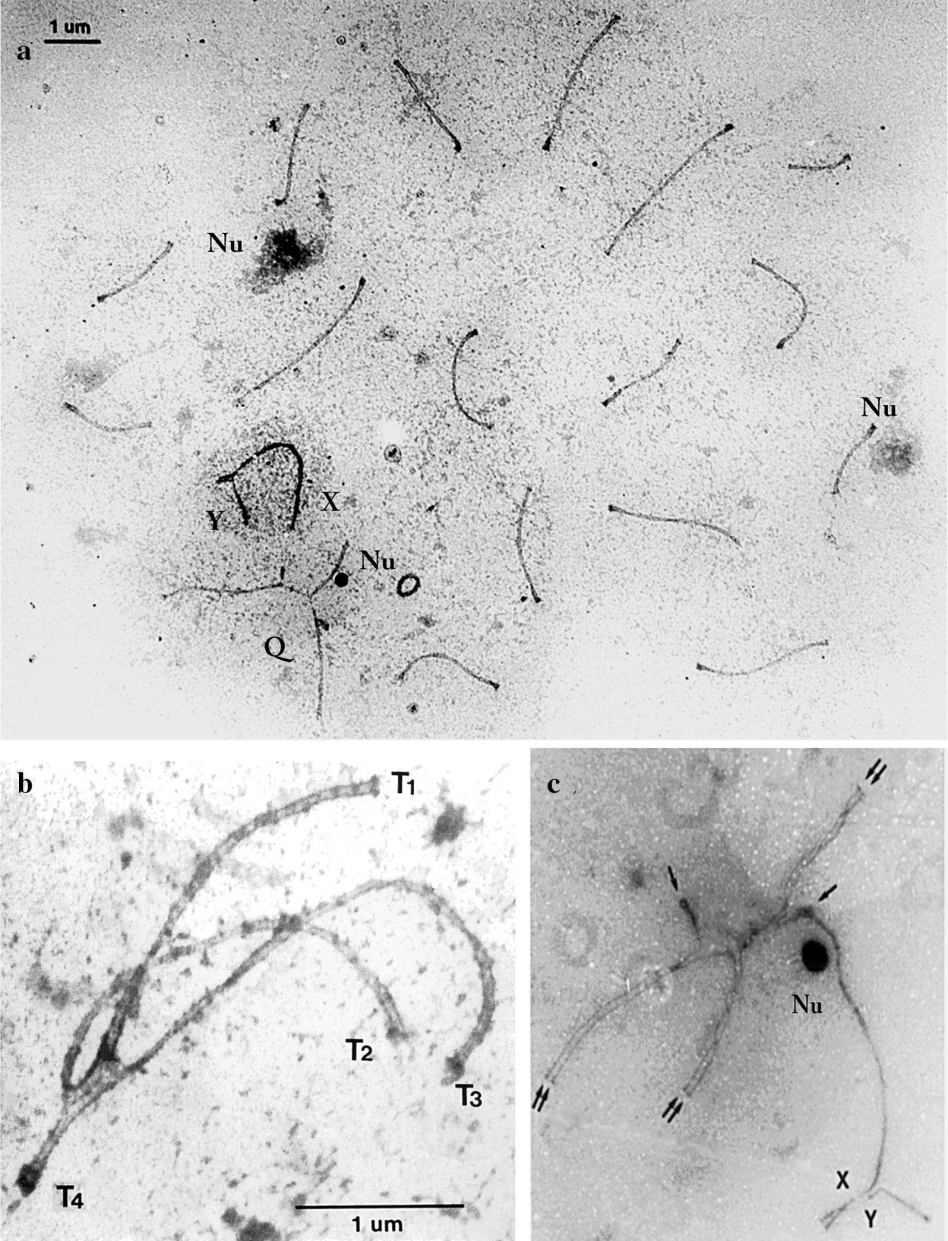
**a** Electron micro spread of a pachytene-spermatocyte nucleus of *Mus domesticus* 2n = 38, double heterozygote for the Rb11.16 and Rb16.17. The synaptonemal complexes of 16 autosomal bivalents, the quadrivalent (Q) and the XY bivalent (XY) are clearly seen. The nucleolar material is also indicated (Nu). Bar = 1 µm. **b** Quadrivalent synaptic configuration. The synaptonemal complex is formed in the whole extension of the four chromosomes, similar to a crosshead. Four synapsed telomeres and 4 points of the quadrivalent’s attachment to the nuclear envelope are also seen. The fourth synapsed telomere also reveals that between the short arms and centromere regions of the non-homologous chromosomes 11 and 17 a synaptonemal complex is formed, where a recombination bar might be present. Telomeres 1, 2, 3 and 4 are indicated. Bar = 1 µm. **c** Unsynapsed quadrivalent associated with the XY bivalent. Electron micro spread showing a thickened single unsynapsed axis of chromosome 11 or 17 connected to the single axis of the X chromosome. Double arrow: well synapsed chromosomes of the quadrivalent; single arrows: single unsynapsed axes of the short arms of chromosomes 11 and 17; XY: sex chromosomes. A dense body of presumed nucleolar material is also present (Nu)

Electron microspreads of pachytene-spermatocytes regularly showed 16 autosomal bivalents, one quadrivalent and the XY bivalent. None of the examined spermatocytes showed univalents or the absence of a quadrivalent. In the quadrivalent, the long arms of the telocentric chromosomes 11 and 17 were synapsed with the respective homologous arms of the Rb metacentric chromosomes; a synaptonemal complex may or may not be formed between the short arms of chromosomes 11 and 17. The X and Y chromosomal axes appeared thicker and synapsed only in a short segment surrounded by condensed chromatin. Frequently electron dense material similar to nucleoli appears connected to two autosomal bivalents and a dense and smaller nucleolus associated with the quadrivalent (Fig. 1a). The detailed analysis of a synapsed quadrivalent showed that the synaptonemal complex is formed in the whole extension of the four chromosomes in terms similar to a crosshead. Although a microspread is a disruptive method where the nuclear organization and the nuclear envelope are broken, nevertheless four synapsed telomeres and therefore the 4 points of quadrivalent attachment to the nuclear envelope can still be seen (Fig. 1b). The fourth synapsed telomere also reveals that synaptonemal complexes can be formed involving the complete short arms and centromere regions of the non-homologous chromosomes 11 and 17, where a recombination bar might be present (Fig. 1b). The length of the synaptonemal complex formed between the proximal non-homologous segments of chromosomes 11 and 17 varied from 0.5 to 1.5 μm in length.

However, although all the quadrivalents studied showed normal synapsis between the homologous chromosomes, many of them showed unsynapsed axes in the non-homologous short arms of telocentric chromosomes 11 and 17. The unsynapsed axes in *cis* or *trans* position were frequently extended to the q chromosomal arms. It was also frequently observed that one of these single axes, lightly thicker than the paired one, was bound to the single axis of the X chromosome (Fig. 1b). Most of these bindings were end-to-end or telomere-to-telomere, although in some spreads the axes involved appeared rather interlocked. In all these associations the X chromosome was otherwise normally paired with the Y chromosome (Fig. 1c). It is not possible to know if the association with the X chromosome was preferential among some of the telocentric chromosomes.

**Fig. 2:**
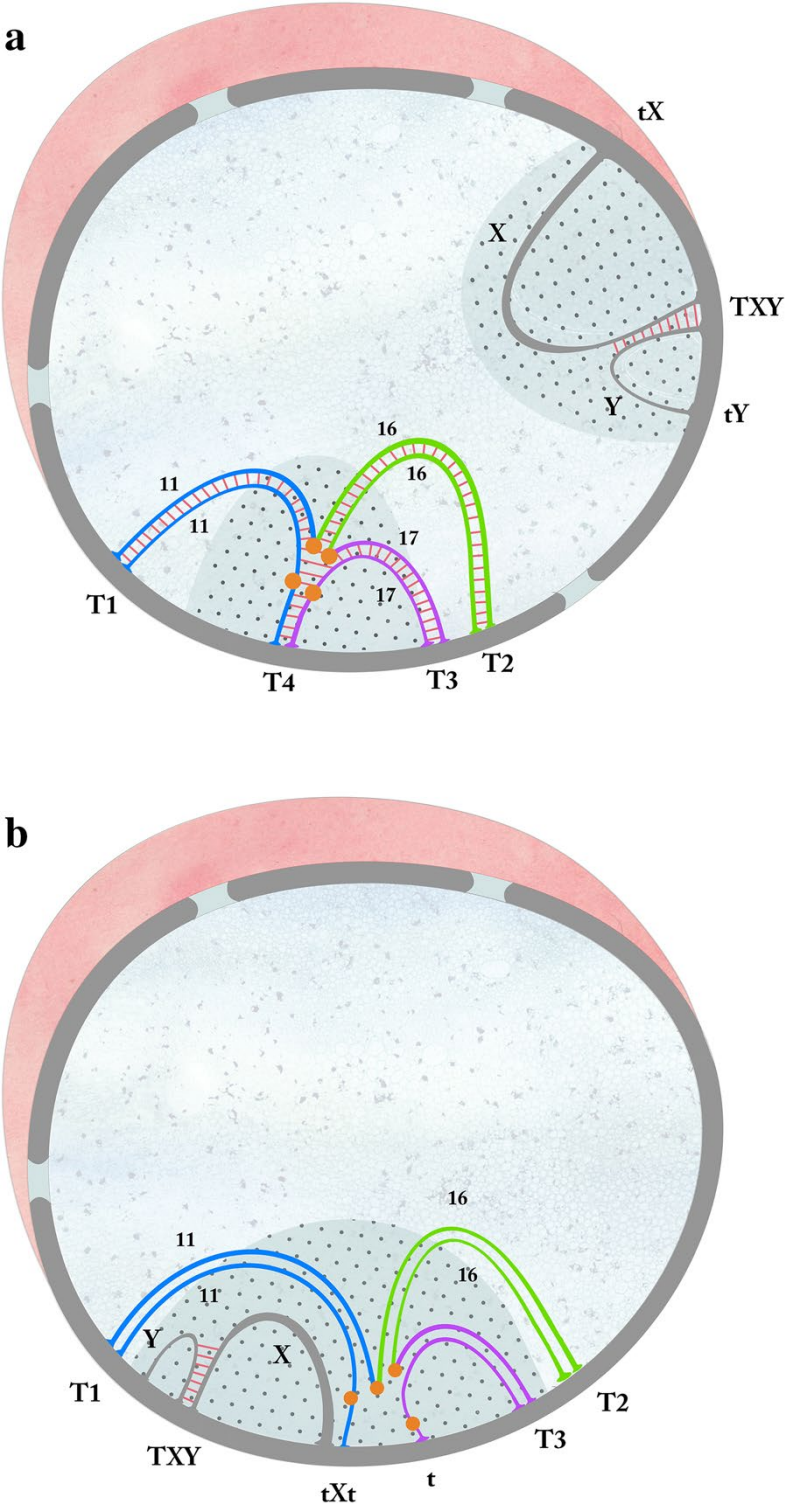
**a** Nuclear configuration of a synapsed quadrivalent not associated with the XY bivalent: the synaptonemal complexes of the chromosomes involved are represented. Chromosomes: 11 in blue, Rb 11.16 in blue and green, Rb 16.17 in green and purple and 17 in purple. The respective centromeres are coloured orange. The quadrivalent’s four synapsed telomeres, all bound to the nuclear envelope, are indicated as T1, T2, T3 and T4. The pericentromeric heterochromatin of the quadrivalent and the condensed sex chromatin are shaded in grey. TXY: region of synapsis between chromosomes X and Y. tX: single axis of the X chromosome; tY: single axis of the Y chromosome. **b** Nuclear configuration of an unsynapsed quadrivalent associated with the XY bivalent. The synaptonemal complexes or single axes of the chromosomes involved are represented. Chromosomes: 11 in blue, Rb 11.16 in blue and green, Rb 16.17 in green and purple and 17 in purple. The respective centromeres are colored orange. The pericentromeric heterochromatin of the quadrivalent is continuous with the condensed sex chromatin, both shaded in grey. The quadrivalent’s three synapsed telomeres (T1, T2, T3) and the single telomeres (t) are independently bound to the nuclear envelope; one of these axes is connected to the single axis of the X chromosome (tX). TXY: region of synapsis between chromosomes X and Y. tY: single axis of the Y chromosome

The nuclear configuration of the synapsed quadrivalent would be especially determined by the fourth telomere, which would drag the centromere regions and heterochromatin of all chromosomes towards the nuclear envelope (Fig. 2a). Notwithstanding, the nuclear configuration of the unsynapsed quadrivalent would be determined by the ectopic association of the single axis with the X chromosome and by the chromatin cluster formed by the pericentromere heterochromatin of the four chromosomes and the condensed XY chromatin, all bound to the nuclear envelope (Fig. 2b).

The quadrivalents of a total of 60 pachytene spermatocyte spreads from different animals were examined by electron microscopy (Table 1). 43% showed total synapsis between the four chromosomes involved. The homologous chromosome arms 11:11, 16:16, 17:17 and the short arms of non-homologous chromosomes 11 and 17 appeared bound through apparently normal synaptonemal complexes. None of the totally synapsed quadrivalents was found to be associated with the XY bivalent. 57% of the spermatocytes showed quadrivalents with unsynapsed axes in the short arms of chromosomes 11 and 17. Most of them (92%) appeared bound to the non-paired axis of the X chromosome. Only 3 of the 34 unsynapsed quadrivalents were not associated with the XY bivalent (Table 1).

**Table 1:** Synapsed or unsynapsed quadrivalents and their binding to the XY chromosomes

Rb heterozygote Male 2n = 38	Examined spermatocytes	Synapsed quadrivalents		Unsynapsed quadrivalents	
		Bound to XY	Not-bound to XY	Bound to XY	Not-bound to XY
Animal 1	16	0	6	8	2
Animal 2	16	0	7	9	0
Animal 3	13	0	5	7	1
Animal 4	15	0	8	7	0
Total	60	0%	26 (43%)	31 (52%)	3 (5%)

## Discussion

### Synapsed quadrivalents

In the quadrivalents studied here, the homologous arms (16) of the two metacentric chromosomes were synapsed, as well as the 11 and 17 arms with the respective q arms of telocentric chromosomes 11 and 17. Also, approximately half of the quadrivalents showed synapses between the short arms (p) of the chromosomes 11 and 17. The configuration of the quadrivalent in the nuclear space appears to be especially determined by the 4th telomere, which would drag all the centromere regions and heterochromatin towards the nuclear envelope (Fig. 2a). This configuration would be similar to that frequently present of three or more telocentric bivalents associated through their pericentromere regions at the nuclear periphery in *Mus* spermatocytes [6]. Although, the heterochromatin in the electron images is not well observed, is clear that a large block of pericentromere heterochromatin coming from all these chromosomes is formed at the nuclear periphery (Fig. 2a). The fourth synapsed telomere stabilizes the *cis* configuration of the quadrivalent, which would favour mechanically normal segregations and possibly chromosomally balanced gametes. In the synapsed configuration the recombination between the short arms of the chromosomes 11 and 17 would also be possible, which might produce genetically unbalanced gametes [7].

The quadrivalent configuration is reiterated in every spermatocyte; the short arms, centromere regions and heterochromatin of chromosomes 11 and 17 are obligatorily together. This repeated convergence of the same pair of heterochromatic domains might be decisive in favouring the formation of a new metacentric Rb chromosome composed of chromosomes 11 and 17. The architectural features together with the intense DNA nicking and repair activity [8] of the first meiotic prophase can set the stage for new chromosome interactions and create the conditions for new chromosome rearrangements [9].

The nucleolar chromosomes of *Mus* 2n = 38 consist of 4 telocentric pairs, 12, 15, 18, and 19, and 2 Rb-derived metacentrics: 11.16 and 16.17 [10]. The derived Rb metacentric chromosomes do not lose the NORs because nucleolar chromosomes in the ancestral *Mus* 2n = 40 bear NORs in the proximal long arm. Electron microscopy of spermatocytes showed abundant nucleolar material beside two telocentrics bivalents and a reduced dense ring beside the quadrivalent. Apparently the expression of NORs originated from the nucleolar telocentrics is different from NORs in nucleolar Rb metacentrics integrated to a quadrivalent. In both cases the nucleolus would occur embedded in a territory formed by heterochromatin, however in quadrivalents the heterochromatin comes from four synapsed chromosomes, which possibly affects nucleolar expression [9].

### Unsynapsed quadrivalents

In the half of the studied quadrivalents the short arms of the chromosomes 11 and 17 and the proximal homologous regions were unsynapsed, possibly due to single telomeres bound very far from or definitely not bound to the nuclear envelope. The unsynapsed axes would be perturbing factors for the multivalent stability, interfering with the normal progression of meiosis [11, 12]. About 90% of these unsynapsed quadrivalents showed a close association with the XY bivalent, particularly with the single telomere of the X chromosome (Fig. 2b). Despite the nuclear disruption produced by the micro spreading method, this association was preserved in such high frequency that it appears to reveal a real and strong union between these chromosomes. That union could arise in the early meiotic prophase when the chromosomes and their heterochromatin are forming a tight cluster in the bouquet configuration [9]. The origin of the association between multivalents and the XY body during pachytene has also been explained as the unavoidable result of the segregation of active and silenced chromatin into separate subnuclear compartments [13]. The main cellular response to synaptic defects is the meiotic silencing of unsynapsed chromosomes (MSUC) that is accomplished by a complex series of epigenetic modifications in the chromatin of the chromosomes involved [14, 15]. The unsynapsed regions of multivalents may trigger MSUC, which may lead to cell apoptosis [16]. Also, the ectopic association of unsynapsed autosomes with the sex chromosomes may result in deregulation of meiotic sex chromosome inactivation (MSCI) [17]. Therefore, the negative effect could be reciprocal, either by the activation of genes on the sex chromosomes, or by the inactivation of genes in the autosomal chromosomes. Any alteration in gene expression patterns would be deleterious for the progression of meiosis, able to trigger cell arrest and apoptosis [18]. Consequently, the unsynapsed quadrivalents and their association with the XY bivalent may be perturbing factors to the progression of meiosis by also altering their normal gene expression [14, 19].

## Conclusions

A quadrivalent is a complex structural organization strongly settled on the nuclear envelope through telomeres and pericentromeric heterochromatin coming from four synapsed chromosomes.

The unsynapsed axes of the heterologous regions bound to the sex chromosomes may be perturbing factors to the progression of meiosis by altering gene expression or chromosome segregation.

A reduced number of spermatocytes, chromosome malsegregation and unbalanced gametes present in Robertsonian heterozygotes may be better understood knowing the configuration that such chromosomal rearrangements may adopt in the first meiotic prophase.

## Methods

### Animals

Four sexually mature 9–12 week-old male mice of *Mus musculus domesticus* double heterozygous for the Robertsonian chromosomes Rb11.16-2H and Rb16.17-32Lub were studied (Additional file 1: Figure S1). The homozygote animals are descendent, through many generations, from animals trapped among feral mice in different sites in Northern Italy. Double heterozygote males were obtained by crossing homozygotes 2n = 39 bearers of metacentric Rb chromosome, which share common chromosome arms (11.16 and 16.17). Mice were fed ad libitum while maintained at 22 °C on a 12/12 h light/dark cycle. The Medical School’s Ethics Committee (CBA #0441) and the Chilean National Science Foundation CONICYT’s Ethics Committee approved the experimental procedures involving mice.

### Electron microscopy of spermatocyte’s spreading

Spermatocyte micro spreads were obtained for electron microscopy (EM) according to the method described by Solari [20]. Briefly, the tubules are torn with fine forceps in Ham F10 without calf serum. The suspension is left in a conical glass centrifuge tube for about 20 min to allow larger fragments to sediment. The supernant containing pachytene cells is changed to another conical tube and centrifuged at 650 rpm for 5 min. Cells were dropped into 0.5% NaCl. Micro spreads were collected on a slide covered with 0.55% polystyrene in chloroform. They were then fixed in 4% paraformaldehyde, 0.03% SDS in sodium borate buffer and contrasted in 1% ethanolic phosphotungstic acid. The grid film was retrieved using parafilm. Selected grids were observed and photographed using a Zeiss EM-109 electron microscope. All images were processed using Adobe Photoshop CS5.1 software or the public domain software ImageJ (National Institutes of Health, United States; http://rsb.info.nih.gov/ij).

## Additional file

**Additional file 1: Figure S1.** Metaphase plate of mitotic chromosomes from a male of Mus domesticus 2n=38, double heterozygote for the Robertsonian chromosomes, Rb 11.16 and Rb 16.17 (Arrows). Giemsa stain.
