# Supplementary figures and images for: Synaptic configuration of quadrivalents and their association with the XY bivalent in spermatocytes of Robertsonian heterozygotes of Mus domesticus
Source: Biol Res. 2017 Nov 23;50:38. doi: 10.1186/s40659-017-0143-6 (PMC5701293; doi:10.1186/s40659-017-0143-6)

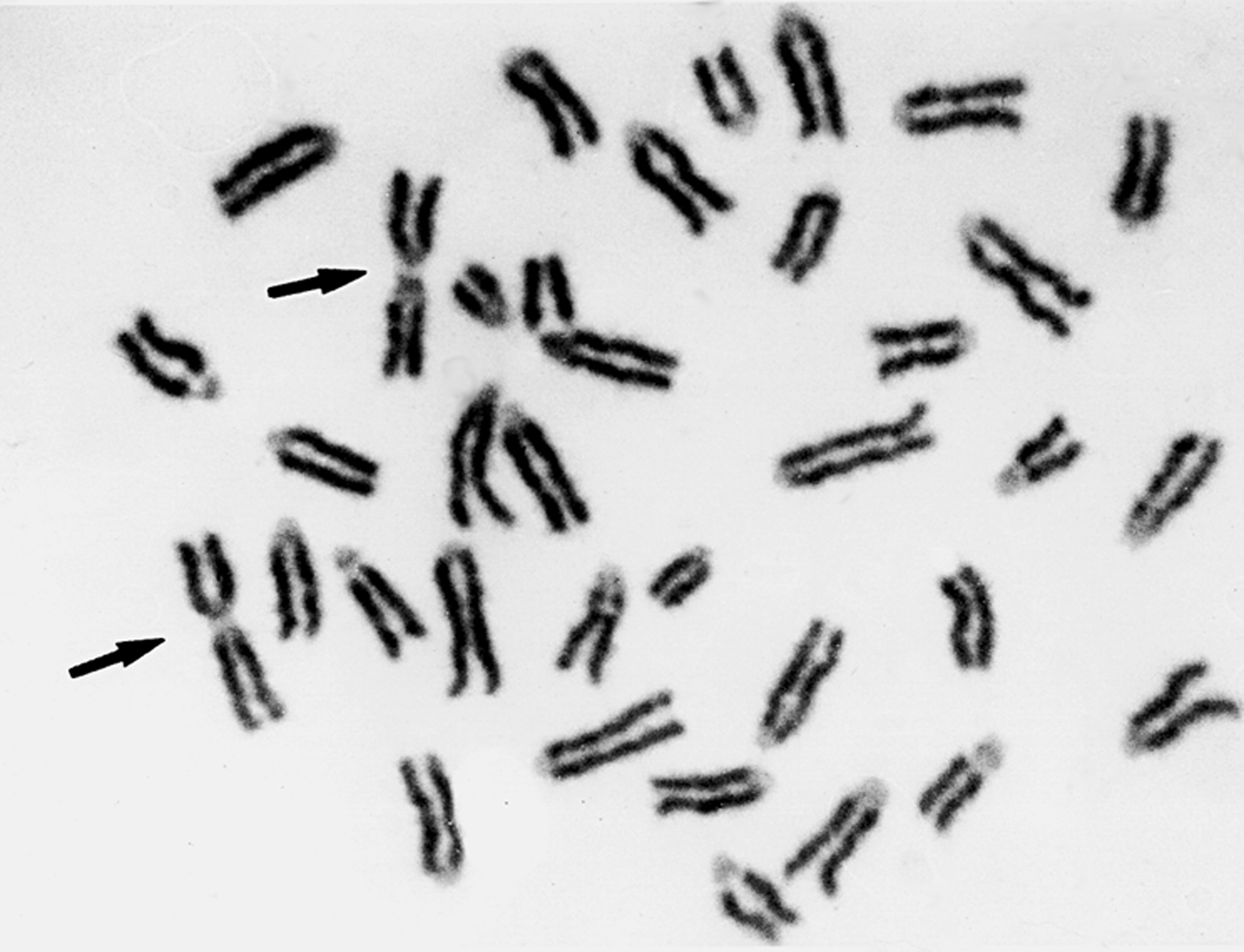

Supplement: Supplementary file 1 — Additional file 1: Figure S1. Metaphase plate of mitotic chromosomes from a male of Mus domesticus 2n=38, double heterozygote for the Robertsonian chromosomes, Rb 11.16 and Rb 16.17 (Arrows). Giemsa stain. [file 40659_2017_143_MOESM1_ESM.jpg]
